# Trans-femoral aortic valve implantation through unrepaired aortic co-arctation

**DOI:** 10.1093/ehjcr/ytad535

**Published:** 2023-10-27

**Authors:** Zaran A Butt, Naeif Almagal, Stephen O’Connor, Mark Hensey

**Affiliations:** Institute of Clinical Trials and Methodology, University College London, Gower Street, London WC1V 6LJ, UK; Department of Cardiology, St. James’ Hospital, Dublin, Ireland; Department of Cardiology, St. James’ Hospital, Dublin, Ireland; Department of Cardiology, St. James’ Hospital, Dublin, Ireland

An 80-year-old female with severe symptomatic aortic stenosis was evaluated for transcatheter aortic valve implantation (TAVI) due to significant surgical risk mainly attributed to underlying severe bronchiectasis (EuroSCORE II 6.19%). Computed tomography (CT) revealed a Sievers type 1 right-non-coronary bicuspid valve (*Panel A*), and aortic diverticulum co-arctation with focal stenosis distal to the left subclavian takeoff (*Panel B*). Minimal luminal diameter was 7.5 mm, which was stable compared to previous imaging. There was no clinical or radiological evidence of secondary complications. Peak-to-peak resting Doppler gradient across the co-arctation was <10 mmHg. The coronary arteries were non-obstructed. Left and right coronary heights were 17 and 12 mm, respectively. Aortic annulus area was 385 mm^2^. A right transfemoral approach was undertaken. A Sentinel cerebral protection device was deployed. A Lunderquist wire was used as a buddy to a JR4 guide catheter to navigate the co-arctation. We successfully tested the passage through the co-arctation with an inflated 12 mm balloon (*Panel C*, Supplementary material online, *Video S1*). The valve was pre-dilated with a 20 mm True balloon (Becton Dickinson) over a Lunderquist wire (Cook Medical). The 23 mm Sapien 3 Ultra Valve (Edwards Lifesciences) was passed over a Safari XS (Boston Scientific) wire (see Supplementary material online, *Video S2*) and implanted successfully (see Supplementary material online, *Video S3*). Post-implantation aortogram revealed a well-seated valve without significant regurgitation (*Panel D*). The post-operative course was unremarkable. Telemetry demonstrated a wandering atrial pacemaker. The patient was asymptomatic and remained well at 30-day follow-up, where electrocardiogram showed sinus rhythm with new first degree atrio-ventricular block (PR 234 ms). Debris collected in the cerebral protection device (*Panel E*) was analysed and determined to be consistent with fibrinous material (*Panel F*). The patient had an excellent clinical and echocardiographic outcome at 30-day follow-up, with surface echo showing a well-seated valve with trivial regurgitation (see Supplementary material online, *Video S4*). Mean valve gradient was 7.9 mmHg. This is only the third reported case of transfemoral TAVI in the setting of an untreated aortic co-arctation. This case demonstrates that traditional access is feasible in such complex cases with meticulous planning and careful patient selection. It also underscores the potential role of cerebral protection devices in patients with complex vasculature and bicuspid valves, who inherently face a higher risk of major vascular complications and neurologic events during TAVI procedures.

**Figure:**
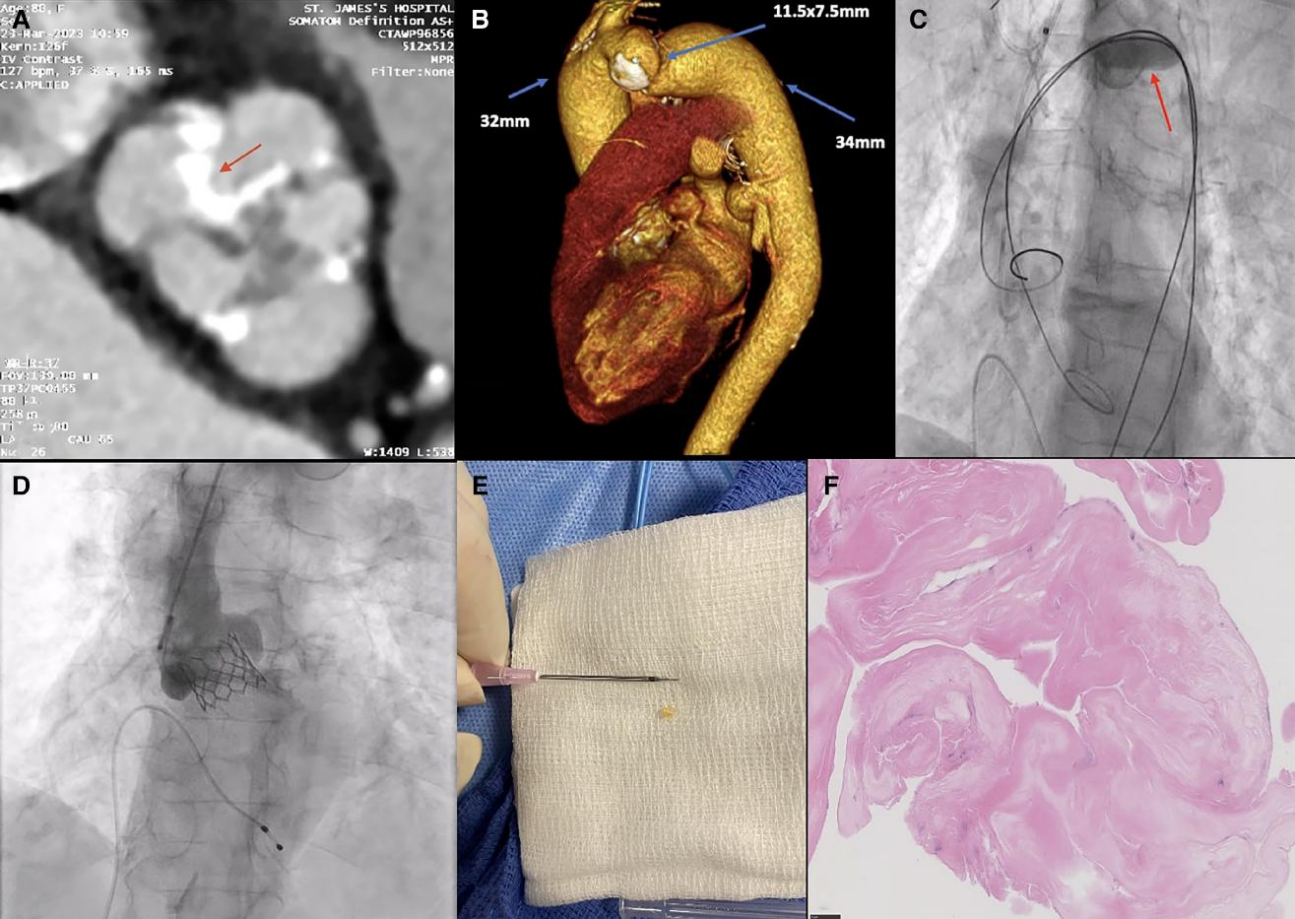


(*Panel A*) Computed tomography demonstrating Sievers type 1 bicuspid aortic valve with single raphe (arrow) between the fused right and non-coronary cusps. (*Panel B*) 3D reconstruction of pre-procedural Multi-Slice CT demonstrating aortic co-arctation with arrows showing pre-stenotic diameter (32 mm), minimum luminal diameters (11.5 × 7.5 mm), and post-stenotic diameter (34 mm). (*Panel C*) Inflated 12 mm balloon (arrow) being used to test passage through the co-arctation. (*Panel D*) Final result after deployment of balloon-expandable valve. (*Panel E*) Clinical image of debris collected within cerebral protection device with 20G needle used to scale. (*Panel F*) Histopathological examination with 50 µm scale (bottom left) of collected tissue showing amorphous eosinophilic material in keeping with fibrin.

## Supplementary Material

ytad535_Supplementary_Data

## Data Availability

All data relevant to this report is included in this manuscript.

## Supplementary material

**Consent**: Written informed consent was obtained from the patient for publication and presentation of this work.

**Funding:** No specific funding was sought for this work.

